# Extraction Kinetics and Reaction Rates of Sacred Lotus Stamen Tea Infusion-Derived Flavonoids in Relation with Its Antioxidant Capacity

**DOI:** 10.3390/plants11172234

**Published:** 2022-08-29

**Authors:** Duangjai Tungmunnithum, Laurine Garros, Samantha Drouet, Natália Cruz-Martins, Christophe Hano

**Affiliations:** 1 Department of Pharmaceutical Botany, Faculty of Pharmacy, Mahidol University, Bangkok 10400, Thailand; 2 Department of Chemical Biology, Eure et Loir Campus, University of Orleans, 28000 Chartres, France; 3 Le Studium Institue for Advanced Studies, 1 Rue Dupanloup, 45000 Orléans, France; 4 Faculty of Medicine, University of Porto, 4200-319 Porto, Portugal; 5 Institute for Research and Innovation in Health (i3S), University of Porto, 4200-319 Porto, Portugal; 6 Institute of Research and Advanced Training in Health Sciences and Technologies (CESPU), Rua Central de Gandra, 1317, 4585-116 Gandra PRD, Portugal; 7 TOXRUN—Toxicology Research Unit, University Institute of Health Sciences, CESPU, CRL, 4585-116 Gandra, Portugal

**Keywords:** *Nelumbo nucifera* Gaertn., tea, stamen, infusion time, flavonoids, antioxidant, extraction kinetics

## Abstract

Stamen tea from *Nelumbo nucifera* Gaertn. (or the so-called sacred lotus) is widely consumed, and its flavonoids provide various human health benefits. The method used for tea preparation for consumption, namely the infusion time, may affect the levels of extractable flavonoids, ultimately affecting their biological effects. To date, there is no report on this critical information. Thus, this study aims to determine the kinetics of solid liquid extraction of flavonoid from sacred lotus stamen using the traditional method of preparing sacred lotus stamen tea. Phytochemical composition was also analyzed using high-performance liquid chromatography (HPLC). The antioxidant potential of stamen tea was also determined. The results indicated that the infusion time critically affects the concentrations of flavonoids and the antioxidant capacity of sacred lotus stamen tea, with a minimum infusion time of 5–12 min being required to release the different flavonoids from the tea. The extraction was modeled using second order kinetics. The rate of release was investigated by the glycosylation pattern, with flavonoid diglycosides, e.g., rutin and Kae-3-Rob, being released faster than flavonoid monoglycosides. The antioxidant activity was also highly correlated with flavonoid levels during infusion. Taken together, data obtained here underline that, among others, the infusion time should be considered for the experimental design of future epidemiological studies and/or clinical trials to reach the highest health benefits.

## 1. Introduction

Polyphenols are plant-based non-nutrients, with remarkable antioxidant effects, naturally occurring in a variety of fruits, vegetables, and seeds that are consumed on a daily basis [1,2,3,4,5,6,7,8,9,10]. Flavonoids are among the most common and widely consumed health-promoting plant polyphenols [8,9,10,11,12,13,14,15], being also of primary importance because of their positive effects on health, not only limited to their functions as natural antioxidants [10], but also their documented roles in the prevention and treatment of cancer [12,14,16], inflammatory, cardiovascular, and neurodegenerative diseases [17]. Lower risks of chronic and age-related degenerative diseases have been linked to their intake from various fruit, vegetables, seeds, and nuts [11,14,18]. Additionally, they have a diverse range of usages as food supplements, pharmaceuticals, or cosmetic ingredients [10,11,12,14,18,19,20,21].

**Figure 1:**
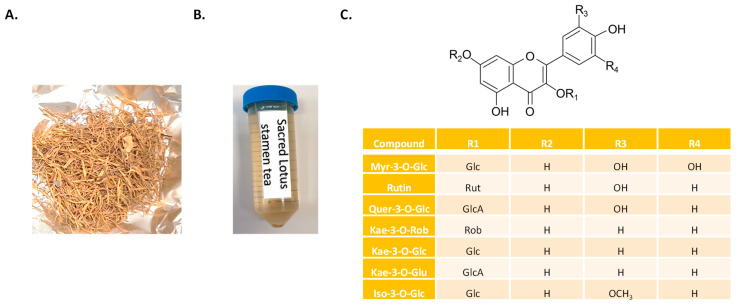
Pictures of sacred lotus (*N. nucifera*) stamen (**A**) and tea obtained after traditional infusion (**B**) and chemical structures of its main flavonoids (**C**) Myr-3-O-Glc: myricetin-3-O-glucose; Quer-3-O-Rut: quercetin-3-O-rutinoside (rutin); Quer-3-O-GlcA: quercetin-3-O-glucuronic acid; Kae-3-O-Rob: kaempferol-3-O-robinobiose; Kae-3-O-Glc: kaempferol-3-O-glucose; Kae 3-O-GlcA: kaempferol 3-O-glucuronic acid; Iso-3-O-Glc: isorhamnetin-3-O-glucose; Glc: glucose; GlcA: glucuronic acid; Rob: robinobiose.

In many Asian countries including Thailand, Japan, China, India, and Sri Lanka, sacred lotus (*Nelumbo nucifera* Gaertn., Nelumbonaceae) is used for popular herbal teas and traditional medicines [12,20,21,22,23,24,25,26,27,28,29,30,31,32,33,34,35]. This aquatic medicinal plant has a long history of use in foods and traditional medicines, in particular in the form of herbal teas [18,20,25,34,35,36,37]. The flower part, and especially the stamen, is the most often used for the preparation of herbal tea as well as for traditional medicine (Figure 1A,B). Sacred lotus stamen tea is widely consumed and provides a variety of beneficial health effects, including improved blood circulation, boosted immune systems, and other biological processes that support human health promotion [18]. 

The stamen of sacred lotus is an abundant source of flavonoid glycosides with potent antioxidant activity [18,20,21,36], including kaempferol (Kae), quercetin (Que), myricetin (Myr), and isorhamnetin (Iso) derivatives (Figure 1C). However, despite being the most important components of herbal teas and traditional medicines, sacred lotus stamen has received little attention. Thus, it is crucial to deepen knowledge on how tea preparation affects the extractability of various health-promoting phytoconstituents, ultimately compromising their medicinal potentialities. The extraction of polyphenols from tea beverages depends critically on the stirring and steeping conditions, including the time of infusion [38,39]. Traditionally, loose stamens are placed directly into a pot and covered with boiling water to make a stamen tea. The stamens are typically taken out after a few minutes, and the infusion is then consumed. Thus, the method used to prepare sacred lotus stamen tea for consumption (e.g., the infusion time), could affect the levels of extractable flavonoids linked to health benefits. There is currently no information available, although such data would be critical for precisely evaluating the health benefits of sacred lotus stamen tea in epidemiological studies or clinical trials. 

As a result, the focus of this study is to determine the kinetics of solid liquid extraction of flavonoids from sacred lotus stamen as well as its antioxidant potential using the traditional method of preparing sacred lotus stamen tea.

## 2. Results and Discussion

### 2.1. Extraction Kinetics of the Sacred Lotus Stamen Tea-Derived Flavonoids during Infusion

**Figure 2:**
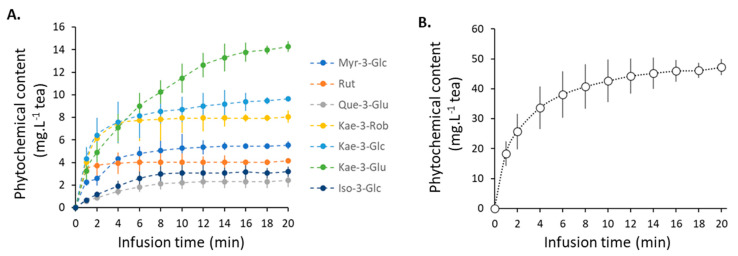
Kinetics of the main individual flavonoids (**A**) and total flavonoids (**B**) from sacred lotus stamen tea infusion.

Sacred Lotus stamen tea contains antioxidant flavonoid glycosides [18,21], including kaempferol (Kae) (Kae-3-GlcA, Kae-3-Glc, and Kae-3-Rob), myricetin (Myr-3-Glc), quercetin (Rutin and Quer-3-Glu), and isorhamnetin (Iso-3-Glc) (Figure 1). All compounds studied in this study are water soluble and are thought to actively contribute to the health-promoting antioxidant effects of sacred lotus stamen tea [18,21]. During infusion, most compounds are rapidly extracted and their concentrations reached a plateau after 5 min, with the exception of Kae-3-GlcA, the main flavonoid extracted from sacred lotus stamen, whose extraction kinetic reached a plateau only after ca. 12 min of infusion (Figure 2A). The release kinetics of these compounds appear to be proportional to their amounts. Because the concentrations reached are all far from their limits of solubility in water (e.g., rutin solubility in water is 125 mg/L, compared to 4.83 mg/L in sacred lotus stamen tea), solubility did not appear to be a limitation parameter. On the other hand, the temperature is known to affect the solubility of flavonoids in water [40]. Here, the temperature dropped from 100 °C to ca. 75 °C during the 20 min infusion, but this parameter did not appear to be critical, as two successive infusions of 10 min with boiled water produced very similar kinetic results (data not shown). Because Kae-3-GlcA is more abundant, the total flavonoid content extraction kinetics logically followed the same trends (Figure 2B). 

### 2.2. Determination of the Reaction Rate of the Flavonoids from Sacred Lotus Stamen Tea during Infusion

**Figure 3:**
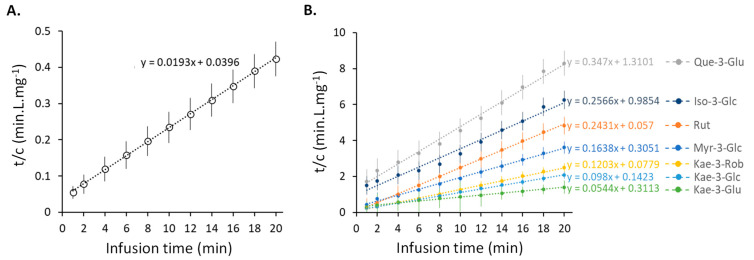
Correlation between t/C and extraction time for total flavonoids (**A**) and the main individual flavonoids (**B**) from sacred lotus stamen tea infusion.

Second=order kinetics were used to fit the concentrations of total flavonoids and each individual flavonoid glycoside that was obtained during the infusion of sacred lotus tea. For each compound under investigation, linear curves (R^2^ > 0.99) for time (t) against time/concentration (t/C) were obtained (Figure 3). It is worth noting that the same second-order kinetics have previously been observed for the extraction of phenolics and caffeine from green tea [39], as well as phenolics from wild olive leaves [41]. The extraction capacity (C_ꚙ_) and the second-order extraction rate (k_1_) were calculated using the slope and intercept of each linear function. These kinetic parameters were determined from data collected during the first 5 min of the infusion under the assumption that the effect of a change in temperature would be minimal. These calculated values of C_ꚙ_ and k_1_, as well as the observed experimental extraction capacity after 20 min of infusion (C_20exp_), are listed in Table 1. Remarkably, the k_1_ values of flavonoid diglycosides (rutin and Kae-3-Rob) were higher than those of the flavonoid monoglycosides. Similarly, the difference between their C_ꚙ_ and the experimental extraction capacity observed 20 min after infusion (C_20exp_) were minimal for these two flavonoid diglycosides when compared to the other flavonoid monoglycosides (Table 1). Because of their higher water solubility, these two compounds may be more effectively extracted. However, no significant differences in C_ꚙ_ and C_20exp_ values were observed, indicating that all individual flavonoids and total flavonoids were efficiently extracted over the 20 min infusion.

### 2.3. Antioxidant Capacity Kinetics of Sacred Lotus Stamen Tea during Infusion 

**Figure 4:**
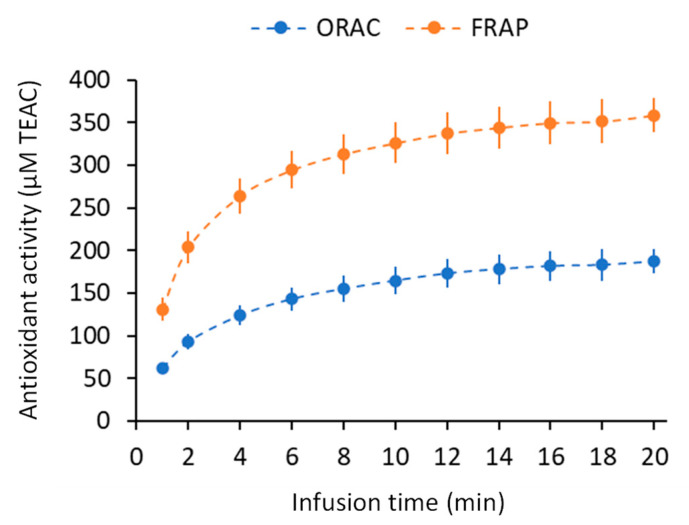
Variation of ORAC and FRAP antioxidant capacity expressed in µM of Trolox-C equivalent Antioxidant Capacity (µM TEAC).

The large part of the medicinal value of sacred lotus stamen tea is attributed to its antioxidant capacity [18]. Here, the ORAC (oxygen radical absorbance capacity) assay and the FRAP (ferric reducing antioxidant power) assay, which rely on antioxidant mechanisms based on hydrogen atom transfer (HAT) and electron transfer (ET), respectively [42], were used to assess the antioxidant capacity kinetics of sacred lotus tea as a function of infusion time. The antioxidant capacity of sacred lotus tea increased with infusion time, reaching a plateau after 12 min (Figure 4). The results suggested that the antioxidant capacity of sacred lotus tea mainly relied on an ET-based mechanism rather than a HAT-based mechanism, with results of FRAP being higher than that of ORAC assay. Similarly, it has been observed that stamen ethanolic extract of *N. nucifera* displayed a high ET-based antioxidant capacity [21]. In addition, our recent work at least suggests that this observed antioxidant capacity of sacred lotus stamen was also obtained in eukaryotic cellular model (yeast cell) [37]. Furthermore, a stronger correlation between the ET-based mechanism and some flavonoids has previously been reported [42,43].

### 2.4. Correlation and Contribution of Each Flavonoid to the Antioxidant Capacity of Sacred Lotus Stamen Tea Infusion

**Figure 5:**
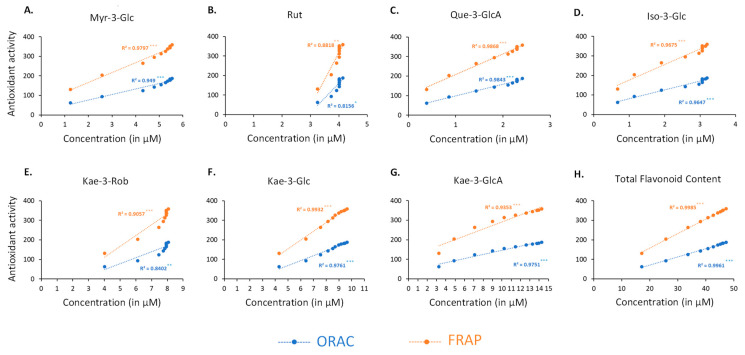
Correlation of antioxidant activity (ORAC (blue line) and FRAP (orange line) assays) with the concentration of Myr-3-Glc (**A**), Rut (**B**), Que-3-GlcA (**C**), Iso-3-Glc (**D**), Kae-3-Rob (**E**), Kae-3-Glc (**F**), Kae-3-GlcA (**G**) and total flavonoids (**H**). Myr-3-O-Glc: myricetin-3-O-glucose; Quer-3-O-Rut: quercetin-3-O-rutinoside (rutin); Quer-3-O-GlcA: quercetin-3-O-glucuronic acid; Kae-3-O-Rob: kaempferol-3-O-robinobiose; Kae-3-O-Glc: kaempferol-3-O-glucose; Kae 3-O-GlcA: kaempferol 3-O-glucuronic acid; Iso-3-O-Glc: isorhamnetin-3-O-glucose; Glc: glucose; GlcA: glucuronic acid; Rob: robinobiose.

Correlation analysis revealed a significant relationship between the antioxidant capacity measured by FRAP (ET-based antioxidant capacity) and the concentration of each flavonoid during sacred lotus stamen tea infusion (Figure 5). 

The highest correlation was found with the FRAP assay when compared to the ORAC assay, confirming the main ET-based antioxidant mechanism. Total flavonoids (R^2^ = 0.9985, *p* < 0.001) and Kae-3-Glc (R^2^ = 0.9932, *p* < 0.001) presented the highest correlation values with the FRAP assay, while rutin (R^2^ = 0.8818, *p* = 0.00139) had the lowest. These results confirmed, for the first time in real conditions using the traditional preparation method, the main drivers for the antioxidant capacity of sacred lotus stamen tea [18,21].

**Figure 6:**
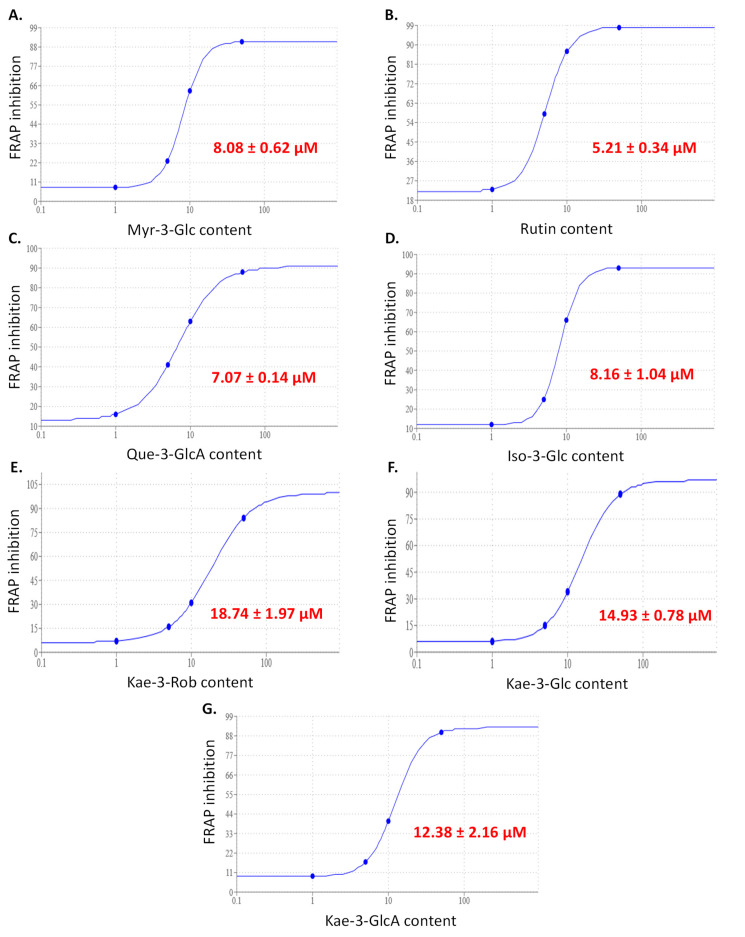
EC50 curves for the FRAP antioxidant activity of Myr-3-Glc (**A**), Rut (**B**), Que-3-Glu (**C**), Iso-3-Glc (**D**), Kae-3-Rob (**E**), Kae-3-Glc (**F**), and Kae-3-Rob (**G**). Myr-3-O-Glc: myricetin-3-O-glucose; Quer-3-O-Rut: quercetin-3-O-rutinoside (rutin); Quer-3-O-GlcA: quercetin-3-O-glucuronic acid; Kae-3-O-Rob: kaempferol-3-O-robinobiose; Kae-3-O-Glc: kaempferol-3-O-glucose; Kae 3-O-GlcA: kaempferol 3-O-glucuronic acid; Iso-3-O-Glc: isorhamnetin-3-O-glucose; Glc: glucose; GlcA: glucuronic acid; Rob: robinobiose.

To evaluate the respective contribution of each flavonoid, the half maximal effective concentration (EC_50_) toward the FRAP assay was calculated (Figure 6). The EC_50_ values ranged from 5.21 to 18.74 µM, with quercetin derivatives outperforming myricetin, isorhamnetin, and kaempferol derivatives. This observation is consistent with the structure–antioxidant activity relationships of flavonoids [42]. The DPPH assay was used to obtain the majority of data available in the literature to classify the antioxidant capacity of flavonoid standards [42]. However, the use of the FRAP assay makes more sense given the present results of the antioxidant assays for the sacred lotus stamen tea. Moreover, the FRAP assay is affordable, the reagents are easy to prepare, the results are highly reproducible, and the process is simple, quick, and highly representative of the biological fluids [44]. Here, rutin was the most powerful antioxidant flavonoid from sacred lotus stamen tea, while Kae-3-Rob was the least effective. However, when compared to widely used food and nutraceutical antioxidants, like Trolox-C [45] or BHT [46], it is worth noting that the EC_50_ values obtained for each flavonoid from sacred lotus stamen tea are in the top range for the FRAP assay. To estimate the relative contribution of each flavonoid, it is also important to consider their respective concentrations. The main flavonoids in sacred lotus stamen tea are kaempferol derivatives, with the Kae-3-GlcA concentration being 3.5 times higher than the rutin concentration (Figure 2). As a result, Kae-3-GlcA is certainly a significant contributor to the antioxidant capacity of sacred lotus stamen tea. This result is consistent with previous studies [21,31], especially those of Jung et al. [24], who were the first to show that kaempferol glycosides are responsible for the radical scavenging potential of *N. nucifera* stamen extract.

## 3. Materials and Methods

### 3.1. Plant Material

Dried sacred lotus stamen tea was purchased from Indian Jadi Booti (Shahdara, India). The lotus stamen that was used in this study comes from the same lots and company that is one of the leaders in the field of Ayurvedic herbal products, which provides genuine herbs in their original forms without artificial material added takes care about storage time, pays attention to the harvesting age of the plant, and processes our herbs as per the traditional/machine methods to preserve their natural quality. We also carefully checked the quality and adulteration [20] of this raw plant material prior to use in this study.

### 3.2. Chemicals and Reagents

All solvents and reagents used for the extraction methodology and the HPLC analysis were of analytical grade or present the highest available purity (Thermo Fischer Scientific, Illkirch, France). Deionized water was purified using the Milli-Q water purification system (Merck Millipore Fontenay sous Bois, Paris, France). Furthermore, all solutions for HPLC analysis were filtered by using 0.45 µm nylon syringe membranes prior to use. The standard compounds were also purchased from Extrasynthese (Genay, France). 

### 3.3. Preparation of Sacred Lotus Stamen Tea

Tea brewing was prepared according to the traditional sacred lotus tea preparation method. Briefly, 500 mL of deionized water was boiled in a glass beaker that was placed on a hot plate. At the onset of boiling, heating was terminated, and 5.0 g of sacred lotus stamen was added into boiled water. Then, the beaker was covered with a watch glass. The magnetic stirrer was used to ensure a constant speed, also maintaining the sample homogeneity. A volume of 1.0 mL of tea was taken after different infusion times: 0, 1, 2, 4, 6, 8, 10, 12, 14, and 20 min, and centrifuged for 5 min at 13,000× *g*. The resulting supernatant was then assayed for flavonoid content, HPLC analysis, as well as antioxidant activity.

### 3.4. Kinetics Evaluation

The solid liquid extraction kinetics of flavonoid phytochemicals were determined. The second-order rate law for the extraction of phytochemicals from stamen tea was measured following the formula used by Chan and his research group [47]:

dc/dt = k_1_(c − c_ꚙ_)^2^ (1)

in which,

k_1_  =  the second-order extraction rate constant (mg/L/min)

c_ꚙ_  =  the extraction capacity (concentration of stamen tea constituents at saturation in mg/L)

c =  the concentration of stamen tea constituents in the solution at any time (mg/L)

t = the infusion time (min)

By considering the boundary condition t  =  0 to t and C  =  0 to C, the integrated rate law for a second-order extraction was determined.

c = (c_ꚙ_^2^.k_1_.t)/(1 + c_ꚙ_.k_1_.t) (2)

By linear transformation of the above equation, the constant rate k_1_ was obtained by fitting the experimental data.

t/c = 1/(k1.c_ꚙ_^2^) + t/c_ꚙ_ (3)

### 3.5. Determination of Total Flavonoid Content (TFC)

The total flavonoid content (TFC) was determined using the aluminum trichloride (AlCl_3_, Sigma Aldrich) colorimetric method, with some modifications [13]. Briefly, 10 μL of the sample was mixed with 10 μL of potassium acetate (1 M), 10 μL of aluminum trichloride (10%, w/v), and 170 µL of double distilled water. The mixture was then incubated at room temperature (25 ± 2 °C) for 30 min. After that, the absorbance was measured at 415 nm by using a UV–visible spectrophotometer (BioTek ELX800 Absorbance Microplate Reader, BioTek Instruments). Quercetin standard (Sigma Aldrich) was used for the calibration curve. TFC was expressed in milligrams of quercetin equivalent/gram.

Total flavonoid production (mg·L^−1^) = DW (g·L^−1^) × TFC (mg·g^−1^).

### 3.6. High-Performance Liquid Chromatography (HPLC) Analysis

In this study, the HPLC system consisting of an autosampler, Varian (Les Ulis, France) Prostar 230 pump, as well as a Varian Prostar 335 photodiode array detector was used, and controlled by Galaxie software (Varian v1.9.3.2). The separation was performed at 35 °C using C18 core-shell column with iso-butyl side chains with TMS endcapping (Kinetex 5 µm XB-C18, 100 Å, LC Column 150 × 4.6 mm, core-shell silica, Phenomenex Le Pecq France). The mobile phase was composed of a methanol (solvent A) and HPLC grade water (solvent B) mixture, both being acidified with 0.05% formic acid. The linear gradient was applied for the mobile phase variation from a 5:95 (*v*/*v*) to a 100:0 (*v*/*v*) mixture of solvents A and B, respectively. Flow rate was 1.30 mL/min, and injection volume was 3 µL. Maximum back pressure was 110 bar. The detection was conducted using a PDA (photodiode array detector), and the quantification was performed at 320 nm (the maximum absorbance wavelength of the studied flavonoids). Flavonoid phytochemicals were then identified by means of comparison with the authentic standards (Extrasynthese, Genay, France), as described previously [36]. 

### 3.7. Antioxidant Oxygen Radical Absorbance Capacity (ORAC) Assay 

The ORAC assay was performed following the procedure used by Prior and his team [48]. In brief, 10 μL of extract was mixed with 190 μL of 0.96 µM fluorescein in a 75 mM phosphate buffer (pH 7.4). After that, the mixtures were incubated at 37 °C for 20 min. Then, 20 µL of 119.4 mM 2,2′-azobis-amidinopropane (ABAP) was added, and the fluorescence intensity was recorded every 5 min for 2.5 h at 37 °C, using a fluorescence spectrophotometer (BioRad, Marnes-la-Coquette, France), which was set with excitation at 485 nm and emission at 535 nm. The ORAC assay was made in triplicate. The results were expressed in terms of the Trolox C-equivalent antioxidant capacity.

### 3.8. Antioxidant Ferric-Reducing Antioxidant Power (FRAP) Assay

The FRAP assay was used following the method used by Benzie and Strain [44], with some modifications. In brief, 10 μL of extract was mixed with 190 μL of the FRAP solution [20 mM FeCl_3_, 10 mM TPTZ, 6H_2_O and 300 mM acetate buffer (pH 3.6) at the ratio 1:1:10 (*v*/*v*/*v*)]. After that, the mixtures were incubated at 25 ± 1 °C for 15 min. The absorbance of the mixtures was determined at 630 nm by using the BioTek ELX800 Absorbance Microplate Reader (BioTek Instruments). The FRAP assays were made in triplicate and the results were expressed in terms of Trolox C-equivalent antioxidant capacity.

### 3.9. Statistical Analysis 

Statistical analyses were performed using the XLSTAT 2019 suite (Addinsoft, Paris, France). Data obtained resulted from at least three independent replicates, and were presented as means and standard deviations. The significant differences were represented as * *p* < 0.05, ** *p* < 0.01, and *** *p* < 0.001. 

## 4. Conclusions

Sacred lotus stamen tea is a popular beverage in Asia with numerous medicinal properties, and, thus, given its wide spectrum of applications, it is critical to understand how tea preparation affects the extractability of the various health-promoting phytoconstituents. The results of this study demonstrated that the infusion time, using the traditional method for preparing sacred lotus stamen tea, greatly affects the concentrations of flavonoids extracted and, consequently, the antioxidant potential. A minimum infusion time of 5–12 min was required to release the different flavonoids from sacred lotus stamen, with flavonoid diglycosides, such as rutin or Kae-3-Rob, being released faster than flavonoid monoglycosides. The antioxidant capacity was found to be highly correlated with flavonoid levels during infusion. The antioxidant assays pointed to a prominent ET-based antioxidant mechanism. In addition to rutin, kaempferol derivatives appeared to contribute significantly to the antioxidant capacity and, thus, to the health benefits of sacred lotus stamen tea. Additionally, for future study in clinical trials, the antioxidant property in vivo level or human cells should be investigated. Taken together, data obtained underline that the preparation method of sacred lotus stamen tea had a significant impact on the flavonoids’ composition and antioxidant capacity, being thus an important factor to be taken into account for the design of future epidemiological studies and/or clinical trials to assess its health-promoting effects. 

## Figures and Tables

**Table 1:** Kinetic parameters for the extraction of the flavonoids from sacred lotus stamen tea infusion.

	C_ꚙ_ (mg/L)	C_20exp_ (mg/L)	k_1_ (mg/L/min)
Quer-3-Glu	2.88 ± 0.13	2.41 ± 0.34	9.20 × 10^−2^ ± 0.21 × 10^−2^
Iso-3-Glc	3.90 ± 0.07	3.19 ± 0.21	6.68 × 10^−2^ ± 0.33 × 10^−2^
Rutin	4.11 ± 0.13	4.14 ± 0.17	1.04 ± 0.23
Myr-3-Glc	6.10 ± 0.05	5.54 ± 0.25	8.79 × 10^−2^ ± 0.14 × 10^−2^
Kae-3-Rob	8.31 ± 0.11	8.06 ± 0.47	1.86 × 10^−1^ ± 0.11 × 10^−1^
Kae-3-Glc	10.20 ± 0.14	9.63 ± 0.52	6.75 × 10^−2^ ± 0.12 × 10^−2^
Kae-3-GlcA	18.38 ± 0.21	14.26 ± 1.97	0.95 × 10^−2^ ± 0.07 × 10^−2^
Total flavonoids	51.81 ± 1.32	47.23 ± 2.34	9.40 × 10^−3^ ± 0.03 × 10^−2^

## Data Availability

All data supporting this study’ findings are included in this article.

## Sample Availability

Available from the authors upon request.

## References

[1] Molnar R., Szabo L., Tomesz A., Deutsch A., Darago R., Raposa B.L., Ghodratollah N., Varjas T., Nemeth B., Orsos Z. (2022). The Chemopreventive Effects of Polyphenols and Coffee, Based upon a DMBA Mouse Model with microRNA and mTOR Gene Expression Biomarkers. Cells.

[2] ALNasser M.N., Mellor I.R., Carter W.G. (2022). A Preliminary Assessment of the Nutraceutical Potential of Acai Berry (*Euterpe* sp.) as a Potential Natural Treatment for Alzheimer’s Disease. Molecules.

[3] Janceva S., Andersone A., Lauberte L., Bikovens O., Nikolajeva V., Jashina L., Zaharova N., Telysheva G., Senkovs M., Rieksts G. (2022). Sea Buckthorn (*Hippophae rhamnoides*) Waste Biomass after Harvesting as a Source of Valuable Biologically Active Compounds with Nutraceutical and Antibacterial Potential. Plants.

[4] Hao J., Lou P., Han Y., Zheng L., Lu J., Chen Z., Ni J., Yang Y., Xu M. (2022). Ultraviolet-B Irradiation Increases Antioxidant Capacity of Pakchoi (*Brassica rapa* L.) by Inducing Flavonoid Biosynthesis. Plants.

[5] Ferrier M., Billet K., Drouet S., Tungmunnithum D., Malinowska M.A., Marchal C., Dedet S., Giglioli-Guivarc’h N., Hano C., Lanoue A. (2022). Identifying Major Drivers of Antioxidant Activities in Complex Polyphenol Mixtures from Grape Canes. Molecules.

[6] Rodríguez-Landa J.F., German-Ponciano L.J., Puga-Olguín A., Olmos-Vázquez O.J. (2022). Pharmacological, Neurochemical, and Behavioral Mechanisms Underlying the Anxiolytic- and Antidepressant-like Effects of Flavonoid Chrysin. Molecules.

[7] Lombardo S., Scavo A., Pandino G., Cantone M., Mauromicale G. (2022). Improvement in the Cynaropicrin, Caffeoylquinic Acid and Flavonoid Content of Globe Artichokes with Gibberellic Acid Treatment. Plants.

[8] Di Pierro E.A., Franceschi P., Endrizzi I., Farneti B., Poles L., Masuero D., Khomenko I., Trenti F., Marrano A., Vrhovsek U. (2022). Valorization of Traditional Italian Walnut (*Juglans regia* L.) Production: Genetic, Nutritional and Sensory Characterization of Locally Grown Varieties in the Trentino Region. Plants.

[9] Costea L., Chițescu C.L., Boscencu R., Ghica M., Lupuliasa D., Mihai D.P., Deculescu-Ioniță T., Duțu L.E., Popescu M.L., Luță E.-A. (2022). The Polyphenolic Profile and Antioxidant Activity of Five Vegetal Extracts with Hepatoprotective Potential. Plants.

[10] Hano C., Tungmunnithum D. (2020). Plant Polyphenols, More than Just Simple Natural Antioxidants: Oxidative Stress, Aging and Age-Related Diseases. Medicines.

[11] Nayak B., Liu R.H., Tang J. (2015). Effect of Processing on Phenolic Antioxidants of Fruits, Vegetables, and Grains—A Review. Crit. Rev. Food Sci. Nutr..

[12] Chen D., Daniel K.G., Kuhn D.J., Kazi A., Bhuiyan M., Li L., Dou Q.P. (2004). Green tea and tea polyphenols in cancer prevention. Front. Biosci..

[13] Tungmunnithum D., Drouet S., Kabra A., Hano C. (2020). Enrichment in Antioxidant Flavonoids of Stamen Extracts from *Nymphaea lotus* L. Using Ultrasonic-Assisted Extraction and Macroporous Resin Adsorption. Antioxidants.

[14] Tungmunnithum D., Thongboonyou A., Pholboon A., Yangsabai A. (2018). Flavonoids and Other Phenolic Compounds from Medicinal Plants for Pharmaceutical and Medical Aspects: An Overview. Medicines.

[15] Tungmunnithum D., Drouet S., Garros L., Hano C. (2022). Differential Flavonoid and Other Phenolic Accumulations and Antioxidant Activities of *Nymphaea lotus* L. Populations throughout Thailand. Molecules.

[16] Younas M., Hano C., Giglioli-Guivarc’h N., Abbasi B.H. (2018). Mechanistic evaluation of phytochemicals in breast cancer remedy: Current understanding and future perspectives. RSC Adv..

[17] Quideau S., Deffieux D., Douat-Casassus C., Pouységu L. (2011). Plant polyphenols: Chemical properties, biological activities, and synthesis. Angew. Chem. Int. Ed..

[18] Tungmunnithum D., Pinthong D., Hano C. (2018). Flavonoids from *Nelumbo nucifera* Gaertn., a Medicinal Plant: Uses in Traditional Medicine, Phytochemistry and Pharmacological Activities. Medicines.

[19] Drouet S., Leclerc E.A., Garros L., Tungmunnithum D., Kabra A., Abbasi B.H., Lainé É., Hano C. (2019). A Green Ultrasound-Assisted Extraction Optimization of the Natural Antioxidant and Anti-Aging Flavonolignans from Milk Thistle *Silybum marianum* (L.) Gaertn. Fruits for Cosmetic Applications. Antioxidants.

[20] Tungmunnithum D., Renouard S., Drouet S., Blondeau J.-P., Hano C. (2020). A Critical Cross-Species Comparison of Pollen from *Nelumbo nucifera* Gaertn. vs. Nymphaea lotus L. for Authentication of Thai Medicinal Herbal Tea. Plants.

[21] Tungmunnithum D., Drouet S., Hano C. (2022). Phytochemical Diversity and Antioxidant Potential of Natural Populations of *Nelumbo nucifera* Gaertn. throughout the Floristic Regions in Thailand. Molecules.

[22] Lee J.S., Shukla S., Kim J.A., Kim M. (2015). Anti-angiogenic effect of *Nelumbo nucifera* leaf extracts in human umbilical vein endothelial cells with antioxidant potential. PLoS ONE.

[23] Kim S., Hong K.-B., Jo K., Suh H.J. (2021). Quercetin-3-O-glucuronide in the Ethanol Extract of Lotus Leaf (*Nelumbo nucifera*) Enhances Sleep Quantity and Quality in a Rodent Model via a GABAergic Mechanism. Molecules.

[24] Jung H.A., Kim J.E., Chung H.Y., Choi J.S. (2003). Antioxidant principles of *Nelumbo nucifera* stamens. Arch. Pharm. Res..

[25] Dezhi F., Wiersema J.H. (2001). Nelumbo nucifera. Flora of China.

[26] Deng J., Fu Z., Chen S., Damaris R.N., Wang K., Li T., Yang P. (2015). Proteomic and Epigenetic Analyses of Lotus (*Nelumbo nucifera*) Petals Between Red and White cultivars. Plant. Cell Physiol..

[27] Cho S., Cho H.W., Woo K.W., Jeong J., Lim J., Park S., Seo M., Lim S. (2019). *Nelumbo nucifera* Receptaculum Extract Suppresses Angiotensin II-Induced Cardiomyocyte Hypertrophy. Molecules.

[28] Chen H., Sun K., Yang Z., Guo X., Wei S. (2018). Identification of Antioxidant and Anti- α -amylase Components in Lotus (*Nelumbo nucifera*, Gaertn.) Seed Epicarp. Appl. Biochem. Biotechnol..

[29] Zhu M., Liu T., Zhang C., Guo M. (2017). Flavonoids of Lotus (*Nelumbo nucifera*) Seed Embryos and Their Antioxidant Potential. J. Food Sci..

[30] Zhu M.Z., Wu W., Jiao L.L., Yang P.F., Guo M.Q. (2015). Analysis of flavonoids in lotus (*Nelumbo nucifera*) leaves and their antioxidant activity using macroporous resin chromatography coupled with LC-MS/MS and antioxidant biochemical assays. Molecules.

[31] Temviriyanukul P., Sritalahareuthai V., Promyos N., Thangsiri S., Pruesapan K., Srinuanchai W., Nuchuchua O., Siriwan D., On-nom N., Suttisansanee U. (2020). The Effect of Sacred Lotus (*Nelumbo nucifera*) and Its Mixtures on Phenolic Profiles, Antioxidant Activities, and Inhibitions of the Key Enzymes Relevant to Alzheimer’s Disease. Molecules.

[32] Shen-Miller J., Mudgett M.B., Schopf J.W., Clarke S., Berger R. (1995). Exceptional Seed Longevity and Robust Growth: Ancient Sacred Lotus from China. Am. J. Bot..

[33] Sheikh S.A. (2014). Ethno-medicinal uses and pharmacological activities of lotus (*Nelumbo nucifera*). J. Med. Plants Stud..

[34] Rai S., Wahile A., Mukherjee K., Saha B.P., Mukherjee P.K. (2006). Antioxidant activity of *Nelumbo nucifera* (sacred lotus) seeds. J. Ethnopharmacol..

[35] Lin H.Y., Kuo Y.H., Lin Y.L., Chiang W. (2009). Antioxidative effect and active components from leaves of lotus (*Nelumbo nucifera*). J. Agric. Food Chem..

[36] Tungmunnithum D., Drouet S., Hano C. (2022). Validation of a High-Performance Liquid Chromatography with Photodiode Array Detection Method for the Separation and Quantification of Antioxidant and Skin Anti-Aging Flavonoids from *Nelumbo nucifera* Gaertn. Stamen Extract. Molecules.

[37] Tungmunnithum D., Drouet S., Hano C. (2022). Flavonoids from Sacred Lotus Stamen Extract Slows Chronological Aging in Yeast Model by Reducing Oxidative Stress and Maintaining Cellular Metabolism. Cells.

[38] Khokhar S., Magnusdottir S. (2002). Total phenol, catechin, and caffeine contents of teas commonly consumed in the United Kingdom. J. Agric. Food Chem..

[39] Fernando C.D., Soysa P. (2015). Extraction Kinetics of phytochemicals and antioxidant activity during black tea (*Camellia sinensis* L.) brewing. Nutr. J..

[40] Ko M.J., Cheigh C.I., Cho S.W., Chung M.S. (2011). Subcritical water extraction of flavonol quercetin from onion skin. J. Food Eng..

[41] Lafka T.I., Lazou A.E., Sinanoglou V.J., Lazos E. (2013). Phenolic extracts from wild olive leaves and their potential as edible oils antioxidants. Foods.

[42] Rice-Evans C.A., Miller N.J., Paganga G., Catherine A.R.-E., Nicholas J.M., George P. (1996). Structure-antioxidant activity relationships of flavonoids and phenolic acids. Free Radic. Biol. Med..

[43] Nazir M., Tungmunnithum D., Bose S., Drouet S., Garros L., Giglioli-Guivarc’h N., Abbasi B.H., Hano C. (2019). Differential Production of Phenylpropanoid Metabolites in Callus Cultures of *Ocimum basilicum* L. with Distinct In Vitro Antioxidant Activities and In Vivo Protective Effects against UV Stress. J. Agric. Food Chem..

[44] Benzie I.F., Strain J.J. (1996). The ferric reducing ability of plasma (FRAP) as a measure of “antioxidant power”: The FRAP assay. Anal. Biochem..

[45] Kicel A., Olszewska M.A. (2015). Evaluation of antioxidant activity, and quantitative estimation of flavonoids, saponins and phenols in crude extract and dry fractions of *Medicago lupulina* aerial parts. Nat. Prod. Commun..

[46] Ooh K.F., Ong H.C., Wong F.C., Sit N.W., Chai T.T. (2014). High performance liquid chromatography profiling of health-promoting phytochemicals and evaluation of antioxidant, anti-lipoxygenase, iron chelating and anti-glucosidase activities of wetland macrophytes. Pharmacogn. Mag..

[47] Chan C.H., Yusoff R., Ngoh G. (2014). Modeling and kinetics study of conventional and assisted batch solvent extraction. Chem. Eng. Res. Des..

[48] Prior R.L., Cao G., Matin A., Sofic E., McEwen J., O’Brien C., Lischner N., Ehlenfeldt M., Kalt W., Krewer G. (1998). Antioxidant capacity as influenced by total phenolic and anthocyanin content, maturity, and variety of Vaccinium species. J. Agric. Food Chem..

